# Supplementary material for: Fe1-xNix Alloy Nanoparticles Encapsulated Inside Carbon Nanotubes: Controlled Synthesis, Structure and Magnetic Properties
Source: Nanomaterials (Basel). 2018 Jul 28;8(8):576. doi: 10.3390/nano8080576 (PMC6116220; doi:10.3390/nano8080576)
Supplement: Supplementary file 1 [file nanomaterials-08-00576-s001.pdf]

$Fe_{1-x}Ni_x$ Alloy Nanoparticles Encapsulated inside Carbon Nanotubes: Controlled Synthesis, Structure and Magnetic Properties

Rasha Ghunaim, Christine Damm, Daniel Wolf, Axel Lubk, Bernd Büchner,

Michael Mertig, Silke Hampel

Supporting information

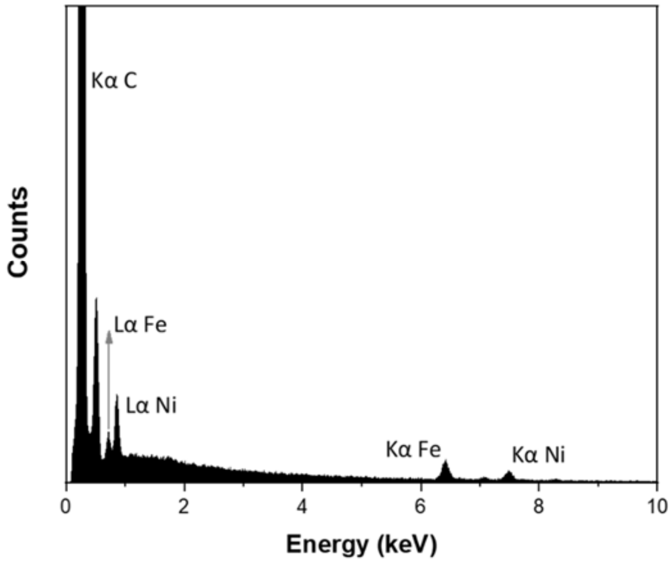

Figure S1: SEM-EDX quantitative measurement for the Fe:Ni ratio over a selected area for a sample of $Fe_{50}Ni_{50}$@CNT, in which the ratio roughly corresponds to 1:1.

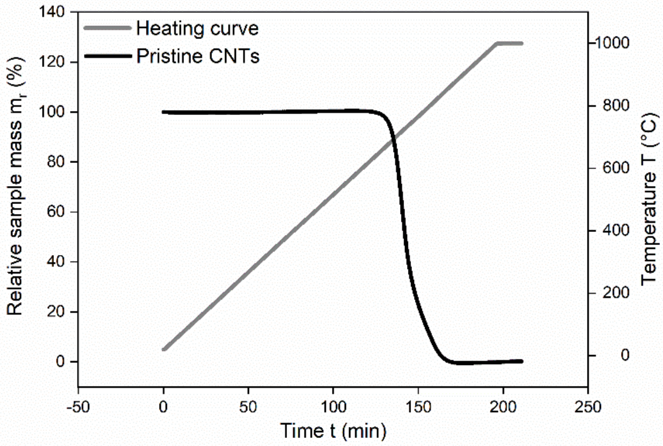

Figure S2: Relative sample mass loss for the pristine CNTs (Pyrograf) during the combustion process, in which 100 % of the CNTs mass has been lost.

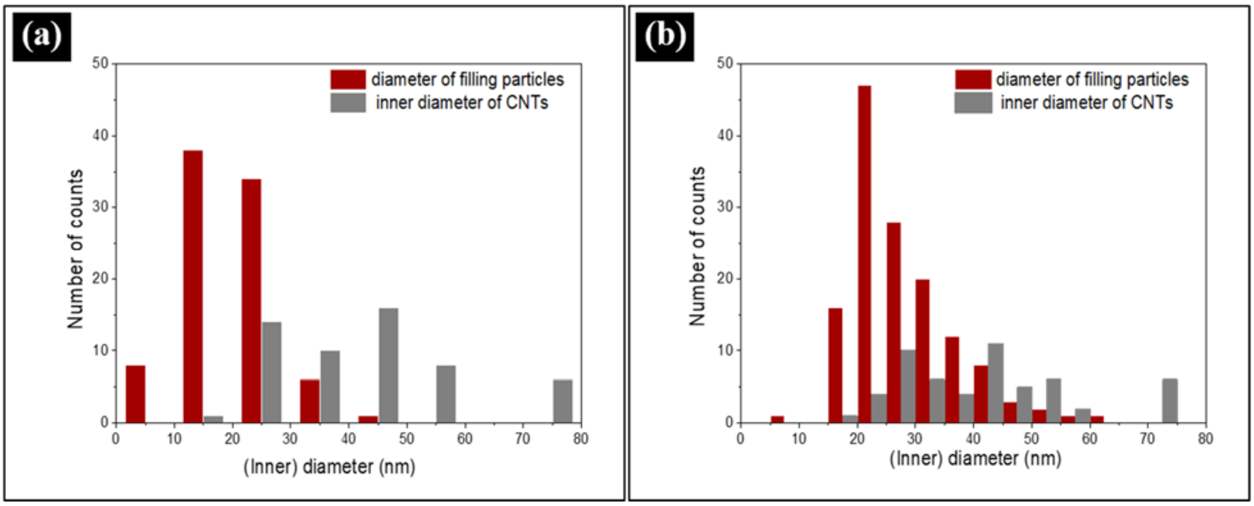

Figure S3: Histograms representing the size distribution of the inner diameter (nm) of CNTs and particles diameters for the a) as-prepared and b) annealed samples of $Fe_{67}Ni_{33}$@CNT.

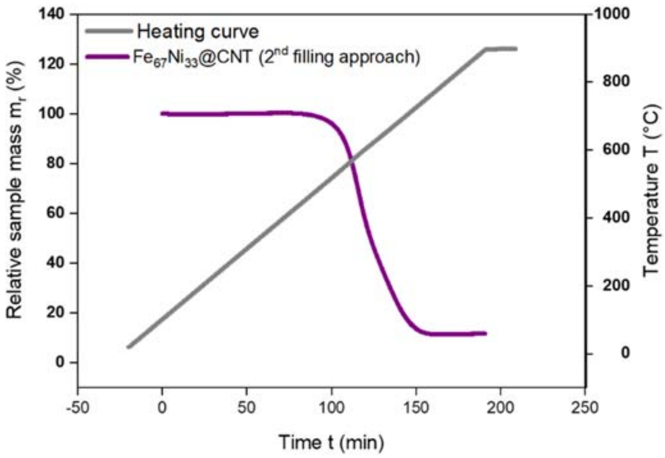

Figure S4: Relative sample mass loss for Fe67Ni33@CNT sample filled by the second approach during the combustion process of the nanocomposite, in which the CNTs mass start to decrease at T ~ 530 °C.
